# Tunable liquid crystal grating based holographic 3D display system with wide viewing angle and large size

**DOI:** 10.1038/s41377-022-00880-y

**Published:** 2022-06-21

**Authors:** Yi-Long Li, Nan-Nan Li, Di Wang, Fan Chu, Sin-Doo Lee, Yi-Wei Zheng, Qiong-Hua Wang

**Affiliations:** 1 School of Instrumentation and Optoelectronic Engineering, Beihang University, Beijing, China; 2 Display Technology Research Center, Seoul National University, Gwanak-gu, Seoul Republic of Korea

**Keywords:** Displays, Liquid crystals

## Abstract

As one of the most ideal display approaches, holographic 3-dimensional (3D) display has always been a research hotspot since the holographic images reproduced in such system are very similar to what humans see the actual environment. However, current holographic 3D displays suffer from critical bottlenecks of narrow viewing angle and small size. Here, we propose a tunable liquid crystal grating-based holographic 3D display system with wide viewing angle and large size. Our tunable liquid crystal grating, providing an adjustable period and the secondary diffraction of the reconstructed image, enables to simultaneously implement two different hologram generation methods in achieving wide viewing angle and enlarged size, respectively. By using the secondary diffraction mechanism of the tunable liquid crystal grating, the proposed system breaks through the limitations of narrow viewing angle and small size of holographic 3D display. The proposed system shows a viewing angle of 57.4°, which is nearly 7 times of the conventional case with a single spatial light modulator, and the size of the reconstructed image is enlarged by about 4.2. The proposed system will have wide applications in medical diagnosis, advertising, education and entertainment and other fields.

## Abstract (web summary)

A tunable liquid crystal grating-based holographic 3D display system is proposed with wide viewing angle and large size.

## Graphical abstract

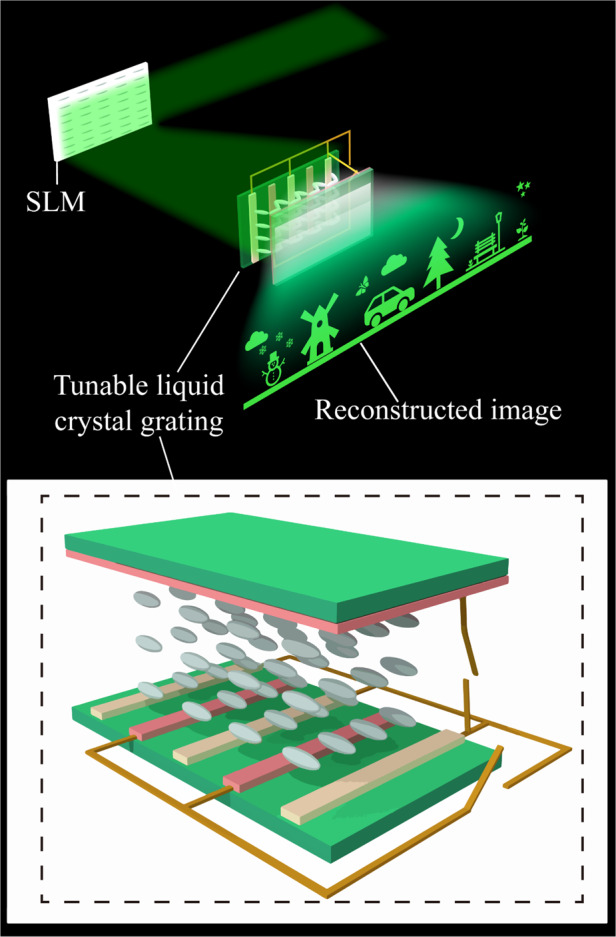

## Introduction

Compared with 2D display, 3D display can present more information, which greatly enhances the interactivity and selectivity of information^1,2^. Holography records and reconstructs the complete wavefront information of the object according to the principles of interference and diffraction^3,4^. Its appearance has promoted the vigorous development of many technologies, such as 3D display, data storage^5,6^, optical encryption^7,8^, medical imaging^9,10^ and digital microscopy^11,12^. As a true 3D display, the holographic 3D display completely avoids the side effects of the traditional 3D display, such as the dizziness and fatigue of viewers. Therefore, it has become the frontier and hotspot of the current research^13,14^. With the proposal of the metaverse concept, the development of the holographic 3D technology has attracted more attention.

Dynamic holographic 3D display with large size and wide viewing angle is the pursuit of people. Dynamic refresh is difficult to achieve with the conventional holographic 3D display technologies based on optical materials such as photorefractive polymers^15,16^. Therefore, the holographic 3D display technology based on the spatial light modulator (SLM) is widely used^17,18^. However, limited by the pixel pitch and size of the SLM, the viewing angle and size of the holographic image are very small. For example, a full parallax 3D image with dimensions of 300 mm × 300 mm × 300 mm and a viewing angle of 30° requires at least 10^12^ pixels on the SLM, which is difficult to achieve even using the state-of-the-art lithography^19^. Currently, the viewing angle of holographic reproduction based on a single SLM is usually less than 9° and the size is less than 2 cm^20,21^.

Various approaches based on time multiplexing^22,23^ or spatial multiplexing^24,25^ of the SLM have been proposed to increase the viewing angle and size of the holographic reproduction. However, the time multiplexing method requires a high refresh rate for the SLM, while the reproduction system structure of the spatial multiplexing method is complex and costly^26^. Since the metasurface structure can achieve subwavelength modulation^27,28^, it is also employed in holographic display systems to expand the viewing angle. Besides, some researchers propose the use of holographic optical elements in the reconstructed system to achieve large size display^29^. Although these methods can effectively expand the viewing angle or enlarge the size, there are some difficulties and challenges in the machining of metasurface structures and holographic optical elements. In 2019, researchers utilized a non-periodic photon sieve to achieve the wide viewing angle holographic reproduction^30^, which presented another idea for the development of the holographic 3D display. Moreover, some researchers introduce aspheric optical systems into holographic systems to expand the viewing angle^31,32^. As a phase-tunable material, in recent years, liquid crystals provide new opportunities for the development of holographic display^33,34^. By using liquid crystal lenses, liquid crystal light valves and other devices, the quality of holographic 3D display is improved. Currently, it is still difficult for a single SLM holographic system to display 3D image with wide viewing angle and large size.

Tunable liquid crystal devices with diverse performances and applications have been demonstrated recently. For example, a stimuliactivated helical cholesteric soft superstructure is proposed, presenting reversible and dynamic transformations between helicidal and heliconical states^35^. Besides, an intrinsic chiral photo-switch with photoreversibility and a switchable helical superstructure system are introduced for facilitating a digitalized selection with a stable laser output^36^. Some researchers design a self-organized 3D soft photonic crystal, which could broaden the application scope of the tunable liquid crystal photonic devices^37^. Moreover, a unique intrinsic chiral photoswitch with broad chirality modulation is proposed to achieve digitally controllable, selectable and extractable multiple stable reflection states^38^. The tunable liquid crystal grating is driven by voltage to realize the adjustment of the light beam, and it can be used in many fields such as optical waveguide, beam deflection, optical interconnects, AR display and 3D display^39–41^. The general optical mechanism of a tunable liquid crystal grating is to generate the periodic electric field distribution in the liquid crystal layer. The electric field is caused by the striped or staggered pattern electrodes on the substrates of the liquid crystal grating, then the grating period can be adjusted by changing the voltage on the electrodes. In 3D display, the tunable liquid crystal grating can be used to improve the display quality. Some researchers use the tunable liquid crystal grating to achieve 2D/3D switching display. When no voltage is applied to the liquid crystal grating, a 2D image can be seen, and a 3D image can be seen when the voltage is applied. The method by using the tunable liquid crystal grating to expand the viewing angle of AR display is also demonstrated.

**Fig. 1:**
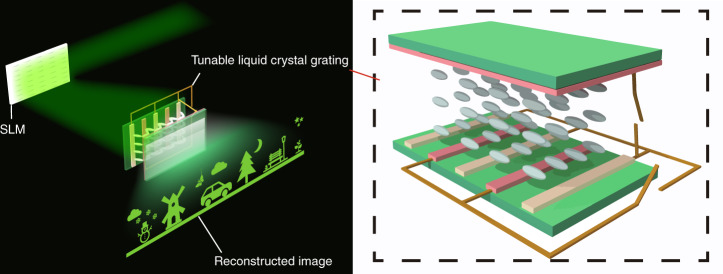
Concept of the proposed system

Here, a new holographic 3D display system based on a tunable liquid crystal grating is proposed, which allows for wide viewing angles and large sizes, as shown in Fig. 1. Different from the traditional holographic system, the liquid crystal grating is placed behind an SLM. The tunable liquid crystal grating (as shown in the dashed box in Fig. 1), providing an adjustable period and the secondary diffraction of the reconstructed image, enables to simultaneously implement two different hologram generation methods in achieving wide viewing angle and enlarged size, respectively. A signal controller is developed in the system to control the switching speed of the hologram and the adjustment of the liquid crystal grating. In the wide viewing angle holographic experiment, the system achieves the viewing angle of 57.4°, which is 7 times that of the conventional system using a single SLM. In the large size holographic reproduction, the system can realize the size magnification of 4.2 times. The proposed system structure is simple and easy to operate, and can also be applied to augmented reality (AR) display.

## Results

### Structure of the proposed system

**Fig. 2:**
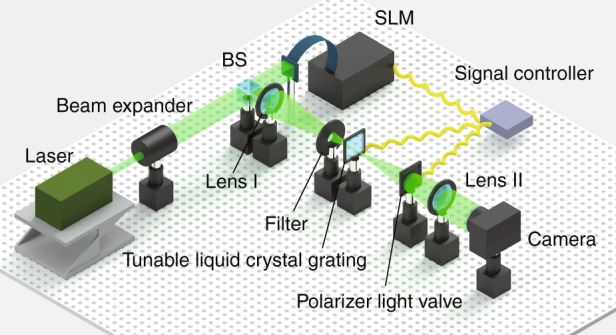
Optical path structure of the proposed system

As shown in Fig. 2, the proposed system consists of a laser, a beam expander, a beam splitter (BS), an SLM, a 4 *f* system (including lens I and lens II), a filter, a tunable liquid crystal grating, a polarized light valve and a signal controller. The laser and beam expander are used to generate the collimated incident light. The collimated incident light irradiates the SLM after passing through the BS. The SLM is loaded with the hologram of the 3D object. The diffracted light passes through lens I after it is reflected by the SLM and BS. The filter is positioned behind lens I and used to eliminate the high-order diffracted light. The tunable liquid crystal grating is located on the back focal plane of lens I and on the front focal plane of lens II. The voltage is applied to the tunable liquid crystal grating to control the diffraction image to generate a secondary diffraction so as to enlarge the viewing angle and size of the holographic 3D display system. The polarized light valve behind the tunable liquid crystal grating is used to control the passage of light with different diffraction orders and adjust the light intensity. A signal control device is designed to generate the hologram and synchronously control the voltage applied to the tunable liquid crystal grating, the loading sequence of the hologram and the state of the polarized light valve. After the secondary diffraction image passes through lens II, the holographic reconstructed image can be captured by a camera.

### Design of the tunable liquid crystal grating

**Fig. 3:**
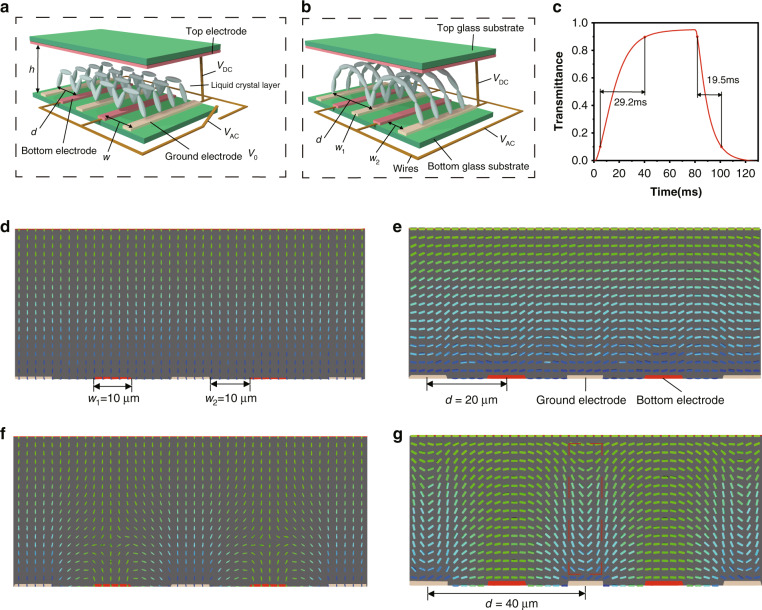
Design of the tunable liquid crystal grating. **a** Distribution of the tunable liquid crystal molecules in the small periodic order. **b** Distribution of the tunable liquid crystal molecules in the large periodic order. **c** Response time of the tunable liquid crystal grating (*w*_1_ = *w*_2_ = 10 μm, the maximum light transmittance is 95%). **d** Intensity distribution of the electric field distribution (*V*_DC_ = 3.7 V, *V*_AC_ = 0 V). **e** Liquid crystal molecular distribution in the small periodic order (*V*_DC_ = 3.7 V, *V*_AC_ = 0 V). **f** Intensity distribution of the electric field distribution (*V*_DC_ = 4 V, *V*_AC_ = 4.5 V). **g** Liquid crystal molecular distribution in the large periodic order (*V*_DC_ = 4 V, *V*_AC_ = 4.5 V). The commercial simulation software: Tech Wiz LCD 3D produced by Sanayi System Company., Ltd., Korea

As shown in Fig. 3, the tunable liquid crystal grating comprises a top glass substrate, a top electrode, a liquid crystal layer, bottom electrodes, a bottom glass substrate and wires. A direct voltage *V*_DC_ is applied to the top electrode and an alternating voltage *V*_AC_ is applied to the bottom electrode. The voltage of the ground electrode is *V*_0_. The width of each bottom electrode is *w*_1_. The gap between the two bottom electrodes is *w*_2_. The distance between centers of the two bottom electrodes is *w* (base pitch). The periodic pitch and the cell gap of the tunable liquid crystal grating are *d* and *h*, respectively. The tunable liquid crystal grating has a fast response speed (Fig. 3c). The period of the grating is adjusted by controlling the magnitude of the *V*_DC_ and *V*_AC_. Thus, the deflection angle of the light rays can be controlled.

When the voltage is only applied to the top electrode (Fig. 3a), the bottom electrode is at zero potential. In this case, the function of the bottom electrode is similar to that of the ground electrode and the electric field distribution around the bottom electrode is similar to that around the ground electrode, as shown in Fig. 3d. The periodic pitch is the same as the base pitch (20 μm) and the distribution of the tunable liquid crystal grating is in the small periodic order, as shown in Fig. 3e. However, when *V*_AC_ is on, the bottom electrode is at a high potential. In this case, the bottom and top electrodes work together and generate a new electric field distribution, as shown in Fig. 3f. The periodic pitch is 40 μm and the distribution of the tunable liquid crystal grating is in the large periodic order, as shown in Fig. 3g.

### Method of wide viewing angle holographic 3D display

**Fig. 4:**
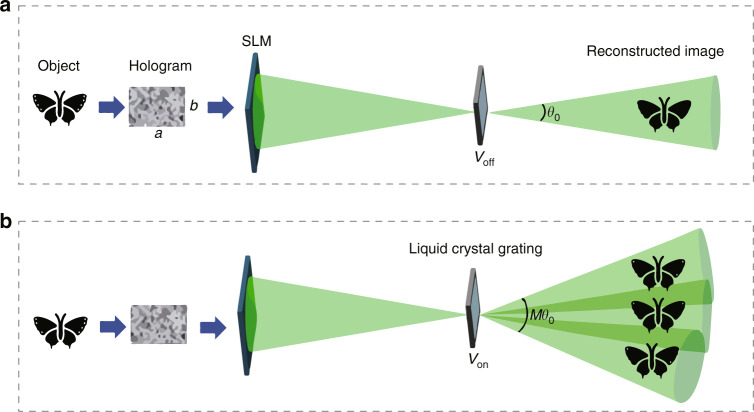
Principle of the wide viewing angle holographic 3D display. **a** Viewing angle of the holographic display in the initial state. **b** Viewing angle when the voltage is applied to the tunable liquid crystal grating

The proposed system realizes wide viewing angle holographic 3D display by using the following method, as shown in Fig. 4. The signal controller is used to generate the hologram of the 3D object. The resolution of the hologram is *a* × *b*. The size of the filter is adjusted to ensure that only the first-order diffraction image can pass through the tunable liquid crystal grating. In the initial state (Fig. 4a), no voltage is applied to the tunable liquid crystal grating and the viewing angle of the system is *θ*_0_. When the voltage is applied to the tunable liquid crystal grating (Fig. 4b), the liquid crystal molecules are arranged in the small periodic order and the diffraction image is subjected to a secondary diffraction. By adjusting the period of the tunable liquid crystal grating, *M* secondary diffraction images can be generated.

Generally, when the planar light wave vertically irradiates a tunable liquid crystal grating, the complex amplitude *E* ~on the grating surface meets Eq. (1):

1 $$\tilde E(x) = \left\{ {\begin{array}{*{20}{c}} {1 + B\cos \frac{{2\pi }}{d}x\;\;\;\;x \le \left| {\frac{{Nd}}{2}} \right|} \\ {0\;\;\;\;\;\;\;\;\;\;\;\;\;\;\;\;\;\;\;\;\;\;x \ge \left| {\frac{{Nd}}{2}} \right|} \end{array}} \right.$$

where the symmetric center of the tunable liquid crystal grating is taken as an origin, *x* indicates the transverse displacement from any point on the grating surface to the origin, *d* indicates the grating pitch, *N* indicates the grating period number, and *B* indicates the peak transmittance of the tunable liquid crystal grating changing with the voltage. The polarization state of the polarized light valve is adjusted, so that the secondary diffraction image can be uniformly displayed in intensity after passing through lens II. Then a wide viewing angle holographic 3D display effect can be achieved, and the viewing angle is *M* × *θ*_0_. The period of the tunable liquid crystal grating can be adjusted by controlling the *V*_DC_ and *V*_AC_ to achieve more flexible wide viewing angle holographic display.

### Method of large size holographic 3D display

**Fig. 5:**
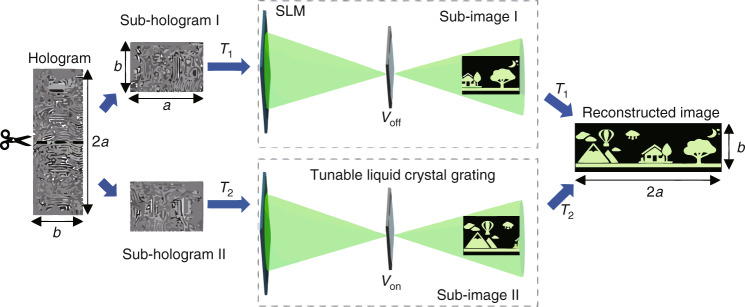
Principle of the large size holographic 3D display

The proposed system realizes large size holographic 3D display by using the following method, as shown in Fig. 5. The resolution of the SLM is *a* × *b*, and the horizontal resolution of the recorded 3D object is *m* (0 < *m* ≤ *a*). Firstly, the signal controller is used to expand the horizontal resolution of the 3D object to *n*, where *n* = *m* + *a*. Then, the hologram of the 3D object is generated with the resolution of 2*a* × *b*. Finally, the hologram is equally divided into two sub-holograms, named as sub-hologram I and sub-hologram II. The resolution of each sub-hologram is *a* × *b*. At moment *T*_1_, sub-hologram I is loaded on the SLM. At this time, the function of the tunable liquid crystal grating is equivalent to that of the transparent glass since no voltage is applied to it. After the diffracted light of sub-hologram I passes through the tunable liquid crystal grating, the polarized light valve, and lens II, the reconstructed image of sub-hologram I can be displayed.

At moment *T*_2_, sub-hologram II is loaded on the SLM by using the signal controller, meanwhile a voltage is applied to the tunable liquid crystal grating to generate the zero-order primary maximum and ±1 order secondary maximum on the spectral plane. The polarized light valve is controlled to ensure that only the positive first-order diffracted light can pass through. The distance *L* between the zero-order primary maximum and ±1 order secondary maximum meets Eq. (2):

2 $$L = \frac{{\lambda f}}{d}$$

where *f* indicates the focal length of lens II, *λ* is the wavelength and *d* is the pitch of the tunable liquid crystal grating. The transverse displacement and the polarization state of the polarized light valve are modified to filter out the zero-order primary maximum and the −1 order secondary maximum. Therefore, only the +1 order secondary maximum can pass through, and the diffraction image of sub-hologram II has a transverse translation. The translation amount is set as *L*, and it equals the transverse size of the SLM:

3 $$L = ap$$

where *p* is the pixel pitch of the SLM.

At moment *T*_2_, the reconstructed image of sub-hologram II can be seen at the +1 order secondary maximum. At moment *T*_1_, the reconstructed image of sub-hologram I can also be seen at the zero-order secondary maximum. When the switching time of *T*_1_ and *T*_2_ is fast enough, the reconstructed images of sub-hologram I and sub-hologram II can be spliced seamlessly in space according to the visual persistence effect of human eyes. Without changing the reconstruction distance and viewing angle, the transverse size of the holographic 3D display can be enlarged to *K* times the original size. *K* meets the following Equation (Supplementary material S1):

4 $$K = \frac{{a + m}}{m}$$

### Reconstruction process

In the experimental system, the wavelength of the laser is 532 nm. The SLM is a reflective phase-only SLM of model FSLM-4K70-P. The pixel pitch of the SLM is 3.74 μm and the resolution is 3840 × 2160. The focal length of the two lenses is 54 cm. The improved novel look up table (NLUT) method is used to generate the hologram. In order to achieve the best display effect with a wide viewing angle, the voltages *V*_DC_ and *V*_AC_ applied to the liquid crystal grating are adjusted in accordance with the resolution of the recorded 3D object, so that the pitch of the liquid crystal grating matches the resolution of the 3D object. If the two do not match, the diffraction images having different orders will interfere with each other or the viewing area will be too sparse to be conducive to view. Therefore, tunable liquid crystal gratings with different pitches are fabricated to match the recorded 3D objects with different resolutions. Here, the parameters of the different liquid crystal gratings are shown in Table 1. The base pitches *w* of the first type and second type tunable liquid crystal gratings are 20 μm and 30 μm, respectively. The effective area width of the prepared liquid crystal grating is 1 cm. The adjustment of the period is accomplished by changing the values of *V*_DC_ and *V*_AC_, and the adjustment of the period is realized. When *w*_1_ = 10 μm, *w*_2_ = 10 μm, the voltage is adjusted to make *d* change from 20 μm to 40 μm, then the period number of the liquid crystal grating can be changed from 50000 to 25000. When *w*_1_ = 15 μm, *w*_2_ = 15 μm, the voltage is adjusted to make *d* change from 30 μm to 60 μm, then the period number of the liquid crystal grating can be changed from 33333 to 16666.

**Table 1:** Parameters of the different tunable liquid crystal gratings

Type	*w*_1_	*w*_2_	*w*	*V*_DC_	*V*_AC_	*d*	Periodic order	*h*
1	10 μm	10 μm	20 μm	3.7 V	0 V	20 μm	Small	10 μm
	10 μm	10 μm	20 μm	4.0 V	4.5 V	40 μm	Large	10 μm
2	15 μm	15 μm	30 μm	3.9 V	0 V	30 μm	Small	10 μm
	15 μm	15 μm	30 μm	4.2 V	4.7 V	60 μm	Large	10 μm

**Fig. 6:**
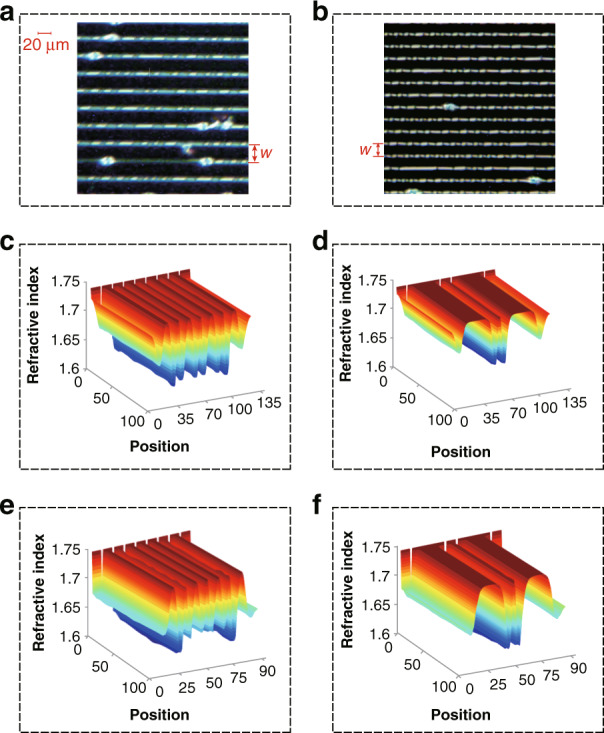
Properties of the tunable liquid crystal grating. **a**, **b** Tunable liquid crystal grating with *w* of 30 μm and 20 μm, respectively. **c, d** Refractive index distribution of the tunable liquid crystal grating with *w* of 30 μm in the small and large periodic orders, respectively. **e, f** Refractive index distribution of the tunable liquid crystal grating with *w* of 20 μm in the small and large periodic orders, respectively

The line focus distributions under the microscope are shown in Fig. 6a, b, and the refractive index distributions of the tunable liquid crystal grating are shown in Fig. 6c–f. The liquid crystal molecules directly above the electrode have deflection and refractive index gradient (shown by the red dotted line in Fig. 3g), so there is a relatively smaller period peak between the larger periods in Fig. 6d, f, but this variation area is very small and it has almost no effect on the quality of the holographic 3D display. In the experiment, the 10 μm liquid crystal grating is used for holographic reproduction. The focusing time of the liquid crystal grating is 29.2 ms, and the dissipation time is 19.5 ms. The process of focusing refers to the transition from no diffraction to maximum diffraction. The dissipation is the recovery of the grating from diffraction maximum to the transparent glass. The diffraction light fields of the first type tunable liquid crystal grating in the small and large periodic order are captured (Supplementary material S2).

**Fig. 7:**
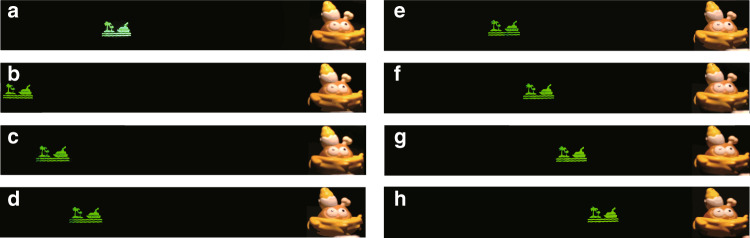
Wide viewing reconstruction of 2D object. **a** Reconstructed image when no voltage is applied to the liquid crystal grating. **b**–**h** Reconstructed image having seven viewing areas

In order to realize the wide viewing angle holographic 3D display, a 2D object ‘island’ is used as the recorded object. The resolution of the recorded object is 1600 × 920 and the reconstructed distance is 15 cm. A hologram with the resolution of 3840 × 2160 is generated and loaded on the SLM by using the signal controller. The recorded object has a transverse resolution of 1600, which fits the small-period mode of the adjustable liquid crystal grating with *w* = 20 μm. When voltage is applied to the tunable liquid crystal grating, a series of secondary diffraction images are generated. By adjusting the voltage, the voltage condition for optimal display is found. When the suitable voltage is applied (the top electrode *V*_DC_ is 3.7 V and the bottom electrode *V*_AC_ is 0 V), seven secondary diffraction images are generated. The polarization state of the polarized light valve is altered, so the intensity of seven secondary diffraction images is uniform. The distance between the reconstructed image and the camera is 50 cm. The real references ‘chick’ is placed at the same depth plane as the image. The camera is placed at the leftmost part of the depth plane and is moved horizontally to the right in this plane until the first secondary diffraction image is captured. Then, the camera is moved further to capture other secondary diffraction images, as shown in Fig. 7. Before a voltage is applied to the tunable liquid crystal grating, the maximum viewing angle of the system is ~8.2° due to the limitation of the SLM. After the second diffraction of the liquid crystal grating, the viewing angle is expanded to ~57.4°.

When no voltage is applied to the liquid crystal grating, the viewing angle is very small, so the camera can only capture reproduced images in this area. When the voltage is applied to the liquid crystal grating, it can be clearly seen that due to the second diffraction, multiple reproduced images are generated, which enlarges the viewing angle by 7 times. Thus, even if the camera moves, the reproduced images can still be captured. It should be noted that holographic 3D display with wide viewing angle can also be achieved under various voltage settings, and the diffraction order can be even higher than seven. For objects with different resolutions, the viewing angle of the reproduced image is guaranteed to be continuous by adjusting the voltage of the liquid crystal grating (Supplementary material Fig. S6). For example, when the large periodic order is used for the object in Fig. 7, it will lead to overlapping of viewports in the reconstructed image (Supplementary material Fig. S7), which seriously affects the quality of the holographic reconstruction. After adjustment, we find that under the voltage conditions in the experiment, the reproduced image intensity can be guaranteed to be displayed uniformly. Thus, the display quality can be ensured.

**Fig. 8:**
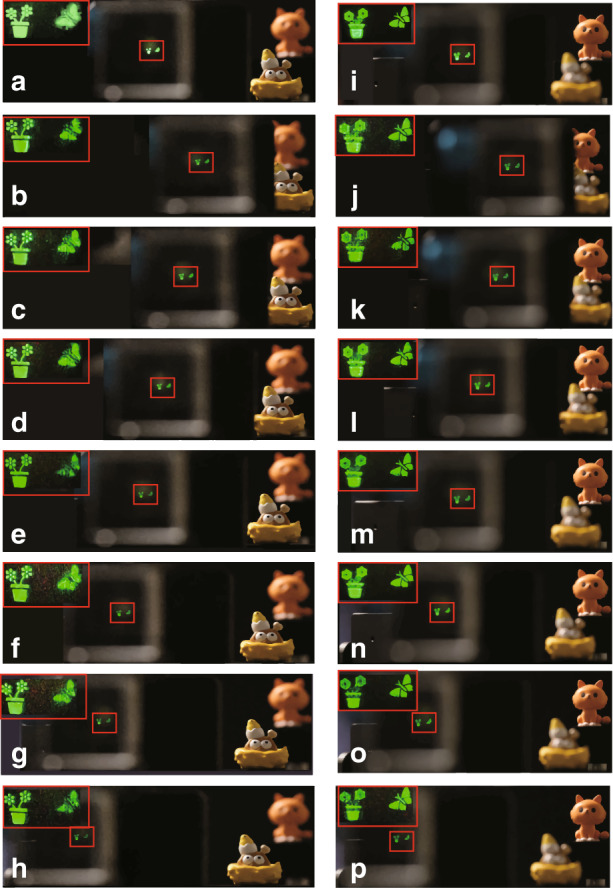
Wide viewing reconstruction of 3D object. **a** Reproduced image of the liquid crystal grating without applied voltage when the ‘flower’ is focused. **b**–**h** Seven diffraction images focused on the ‘flower’ when the voltage is applied to the liquid crystal grating. **i** Reproduced image of the liquid crystal grating without applied voltage when the ‘butterfly’ is focused. **j**–**p** Seven diffraction images focused on the ‘butterfly’ when the voltage is applied to the liquid crystal grating

Furthermore, to verify the 3D holographic display effect, a ‘flower’ and a ‘butterfly’ with different depth information are regarded as the 3D object for hologram recording. The resolution of the 3D object is 1700 × 700. The reconstruction distance of the ‘flower’ and ‘butterfly’ is 15 cm and 25 cm, respectively. As the references, the real objects ‘kitten’ and ‘chick’ are placed at different depths. The ‘kitten’ and ‘butterfly’ have the same depth, as do the ‘flower’ and ‘chick’. The voltage conditions and the images captured method are the same as the 2D holographic display. The experimental results are shown in Fig. 8. Figure 8a is the holographic reproduction of the liquid crystal grating without applied voltage when the ‘flower’ is focused. At this time, only the first-order diffraction image can be seen. Figure 8b–h are the seven diffraction images captured with the movement of the camera when the voltage is applied to the liquid crystal grating. It can be seen that after using the liquid crystal grating, the viewing angle is greatly enlarged. Figure 8i–p are the results when the ‘butterfly’ is focused. To obtain a better view of the details, the image in the red box is magnified. The upper left corner is the enlarged reproduced image. In addition, the dynamic video of the holographic 3D reproduction is captured (Supplementary material S3).

**Fig. 9:**
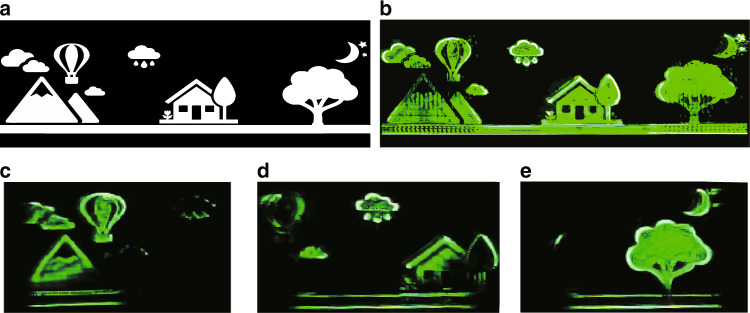
Reconstructed image of the large size holographic display. **a** Complex scene with a resolution of 3840 × 1282. **b** Reconstructed image by using the proposed method. **c** Left marginal viewing area of the reconstructed image by using the traditional method. **d** Middle viewing area of the reconstructed image by using the traditional method. **e** Right marginal viewing area of the reconstructed image by using the traditional method

To realize the large size holographic 3D display, a complex scene is used as the recorded object. The resolution of recorded object is 3840 × 1282 and the reconstruction distance is 10 cm, as shown in Fig. 9a. The resolution of the hologram is 7680 × 2160. In order to solve the problem of long calculation time for ultra-high-resolution hologram, an optimized segmentation method is designed based on the NLUT algorithm (Supplementary material S4). The improved NLUT algorithm is used to generate the holograms and then the generated hologram is divided into two sub-holograms. Each sub-hologram has a resolution of 3840 × 2160.

At moment *T*_1_, sub-hologram I is loaded on the SLM. At this moment, no voltage is applied to the tunable liquid crystal grating, and only the zero-order diffracted light can pass through. At moment *T*_2_, sub-hologram II is loaded on the SLM by using the signal controller, meanwhile a voltage (*V*_DC_ = 3.7 V and *V*_AC_ = 0 V) is applied to the tunable liquid crystal grating. At this time, only the positive first order diffracted light can pass through by controlling the polarized light valve. Compared with sub-hologram I, the diffracted image of sub-hologram II has a transverse translation of 14.364 mm. When the switching time of *T*_1_ and *T*_2_ is fast enough, the diffracted images of sub-hologram I and sub-hologram II are seamlessly spliced in space according to the visual persistence effect of human eyes. Then, a large-sized reconstructed image (Fig. 9b) can be seen.

In order to verify the advantage of the proposed method, the holographic display experiment is performed where the same SLM is used to reconstruct the same complex scene at the same reconstruction distance while using the conventional method. Because the diffraction angle is limited by the pixel pitch of the SLM, the resolution of complex scenes is much higher than the maximum resolution reproduced by the SLM at this reconstruction distance. The camera can only capture part of the reconstructed image. The left and right marginal viewing area holographic display results are shown in Fig. 9c, e, respectively. When the camera is in the middle viewing area, only an incomplete reproduced image can be obtained, as shown in Fig. 9d. Besides, due to the continuous attenuation of the holographic reconstruction light field information at the edge of the viewing area, the reconstruction quality of the edge area is seriously affected. The magnification factor *K* can be calculated from the holographic display results, which is in agreement with the theoretical calculation results of Eq. (3) (*K* = 2).

**Fig. 10:**
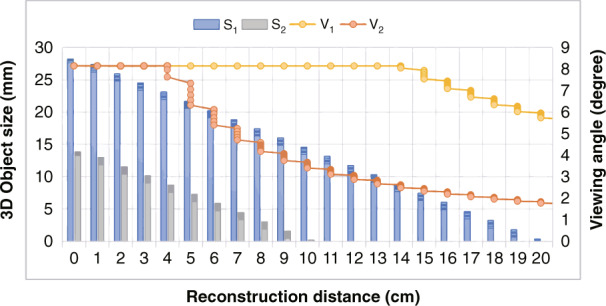
Performance comparison between the proposed large size holographic display method and the traditional method

Furthermore, the simulation experiments are carried out to compare the performance between the proposed and traditional methods, as shown in Fig. 10. *S*_1_ and *S*_2_ are the change histogram of the proposed and traditional methods, respectively. The histogram represents the trend of 3D object maximum size with the reconstruction distance, when the viewing angle equals the limiting diffraction angle. Similarly, the maximum size of the proposed method is larger than that of the traditional method, and the transverse size magnification factor *K* increases rapidly with the increase of the reconstruction distance. The distance of reconstructed image by using the proposed method is nearly twice of the reconstructed image by using the traditional method.

When the transverse resolution of the 3D object is 1200 and the reconstruction distance is 7 cm, *K* = 4.2. So, the proposed method has more advantages in the holographic display with large depth of field. *V*_1_ and *V*_2_ represent the viewing angle change with the reconstruction distance by using the proposed and traditional methods, respectively. It is clear that the proposed method can achieve a wide viewing angle holographic display in longer reconstruction distance. Therefore, the proposed method also has unique advantages in perspective characteristics. In the large size holographic 3D display, the period setting of the liquid crystal grating is related to the lateral size of the SLM and the focal length of the 4 *f* lens. For SLMs and lenses with different parameters, the seamless large-size holographic reproduction can be achieved by adjusting the period of the liquid crystal grating.

## Discussion

The holographic 3D display based on the tunable liquid crystal grating is one of the improved solutions to overcome the restriction of the viewing angle and image size in the current holographic display. By designing the liquid crystal molecular and electrode arrangement, a tunable liquid crystal grating is designed in this paper. The pitch of the tunable liquid crystal grating can be changed by adjusting the voltage applied to the top and the bottom electrodes. The response time of the tunable liquid crystal grating is 29.2 ms, which fully meets the needs of synchronous control. By using the tunable liquid crystal grating, a holographic 3D display system is proposed. Two different hologram generation methods are correspondingly designed to cooperate with the liquid crystal grating to realize the wide viewing angle and large size display, respectively. The system achieves a wide viewing angle holographic display of 57.4°, which is 7 times that of the conventional system using a single SLM. The details of the recorded object are completely reconstructed and the intensity distribution is uniform. The size of the reconstructed image can be enlarged to 4.2 times. The proposed system is expected to be applied in advertising, education, entertainment, and other fields. In the future, we will further consider the colorization of the system and promote the application of the holographic display.

## Materials and methods

### Sample fabrication

During the fabrication of the tunable liquid crystal grating, the inner surface of the top glass substrate is coated with a planar electrode and then a thin alignment material (polyimide, PI) is coated on the inner surface of the top glass substrate for aligning the liquid crystal molecules. The inner surface of the bottom glass substrate is coated with the transparent regular flat strip electrodes. After the PI is spin-coated on the inner surface of the bottom glass substrate, the LC director is homogeneously aligned perpendicular to the direction of the periodic strip electrodes. The top glass substrate alignment material is rubbed in the antiparallel direction with respect to the bottom substrate. When the liquid crystal mixture is injected into the cell, homogeneous alignment is induced by the buffed layers.

## Supplementary information

Supplementary Information for Tunable liquid crystal grating based holographic 3D display system with wide viewing angle and large size

Video m1

Video m2

Video m3

Video m4
