# Supplementary material for: Tunable liquid crystal grating based holographic 3D display system with wide viewing angle and large size
Source: Light Sci Appl. 2022 Jun 21;11:188. doi: 10.1038/s41377-022-00880-y (PMC9213428; doi:10.1038/s41377-022-00880-y)
Supplement: Supplementary file 1 — Supplementary Information for Tunable liquid crystal grating based holographic 3D display system with wide viewing angle and large size [file 41377_2022_880_MOESM1_ESM.docx]

SUPPLEMENTARY MATERIAL

Supplementary Information for : Tunable liquid crystal grating based holographic 3D display system with wide viewing angle and large size

Yi-Long Li^1, †^, Nan-Nan Li^1, †^, Di Wang^1, *^, Fan Chu^1^,

Sin-Doo Lee^2^, Yi-Wei Zheng^1^, Qiong-Hua Wang^1, *^

1*School of Instrumentation and Optoelectronic Engineering, Beihang University, Beijing 100191, China.*

2*Display Technology Research Center, Seoul National University, Gwanak-ro 1, Gwanak-gu, Seoul 08826, Republic of Korea.*

^†^*These authors contributed equally to this work.*

**Correspondence: D Wang, E-mail:* [*diwang18@buaa.edu.cn;*](mailto:diwang18@buaa.edu.cn;)

*QH Wang, E-mail:* [*qionghua@buaa.edu.cn*](mailto:qionghua@buaa.edu.cn)

7 pages, 7 figures S1-S7

**S1: Calculation of the magnification factor *K***

The calculation principle of the magnification factor *K* is shown in Fig. S1. The maximum diffraction angle *α* of the holographic reproduction is equal to the maximum diffraction angle of the SLM, so as to ensure that the viewing angle *β* of the reconstructed image is the largest. For intuitive analysis, the proposed large size holographic 3D display method can be equivalent to two side-by-side SLMs (SLM_1_ and SLM_2_) for holographic reconstruction at the same time. The sizes of SLM_1_ and SLM_2_ are *a*. When the reconstruction distance is *L*, the reconstructed image based on SLM_1_ is image Ⅰ and its size is denoted as *m*. When the large size hologram is loaded on the two SLMs respectively, the increased size can be obtained through the geometric relationship, which is exactly equal to the size of the SLM. Thus, under the condition of maximum viewing angle, *K* can be calculated by Eq. (S1.1),

$K=\frac{a+m}{m}$ (S1.1)

where 0< *m* ≤*a*.


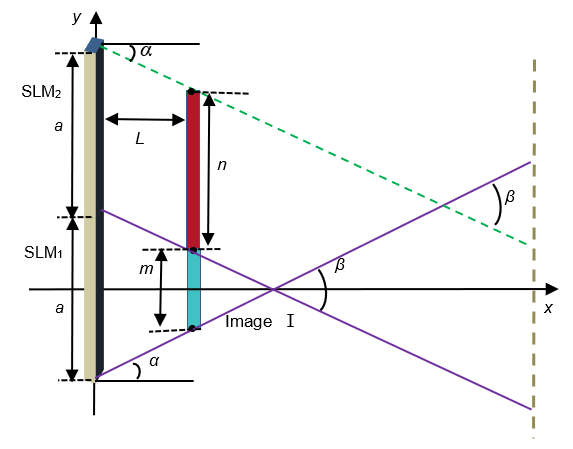


**Figure S1. Calculation principle of the magnification factor *K*.**

**S2:** **Diffraction light field of the tunable liquid crystal grating**

As shown in Fig. S2, the system for capturing the diffraction light field consists of a laser, a polarizer, a tunable liquid crystal grating and a CCD. The wavelength of the laser is 532 nm. The base pitch *w* of the tunable liquid crystal grating is 20 μm. The diffraction light fields of the tunable liquid crystal grating in the small and large periodic order are captured (Video m1 and video m2, respectively). In order to measure the response time of the liquid crystal grating, the liquid crystal grating is placed under an orthogonal polarizer, and the response time of the liquid crystal grating can be calculated by applying a pulse voltage to the liquid crystal grating and measuring the light transmittance.


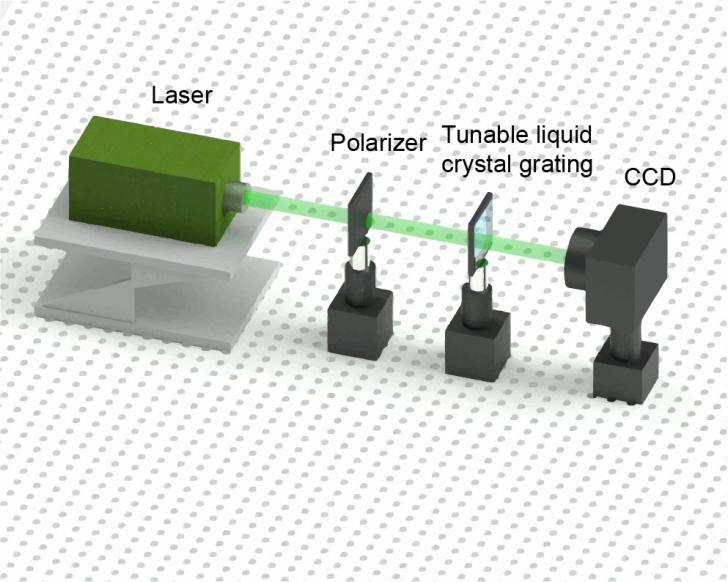


**Figure S2. System for capturing the diffraction light field.**

**S3:** **Dynamic video of the wide viewing angle holographic 3D reproduction**

The dynamic video of the wide viewing angle holographic 3D reproduction is captured. The reconstruction distance of the ‘flower’ and ‘butterfly’ is 15 cm and 25 cm, respectively. As the references, the real objects ‘kitten’ and ‘chick’ are placed at different depths. Among them, the ‘kitten’ and ‘butterfly’ have the same depth, while the ‘flower’ and ‘chick’ have the same depth. The results focused on the ‘flower’ are shown in Figs. S3a-c and Video m3. The results focused on the ‘butterfly’ are shown in Figs. S3d-f and Video m4.


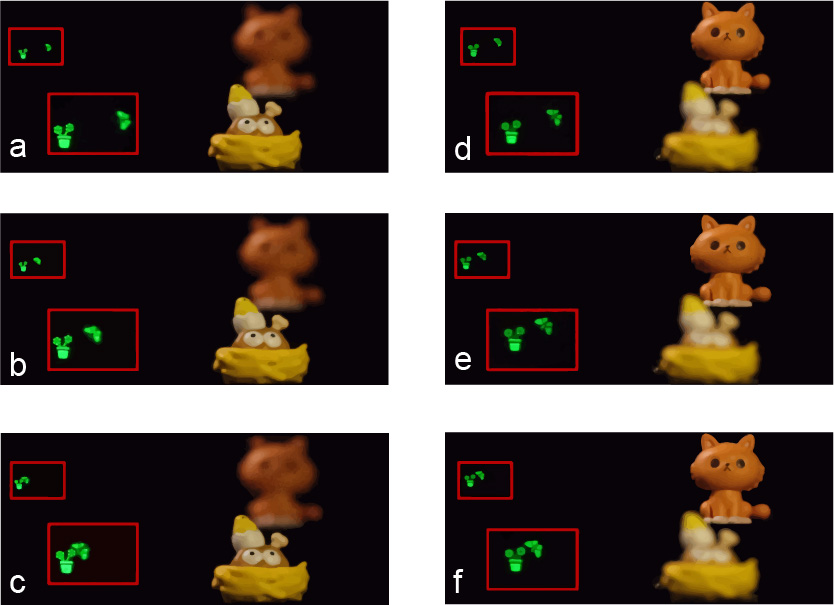


**Figure S3. Dynamic wide viewing reconstruction of 3D object. a-c** Images at different moments focused on the ‘flower’ when the voltage is applied to the liquid crystal grating. **d-f** Images at different moments focused on the ‘butterfly’ when the voltage is applied to the liquid crystal grating.

**S4: Calculation of the hologram in the holographic 3D display system**

In the holographic 3D display system, the optimized segmentation algorithm is designed based on the novel-look-up-table (NLUT) algorithm to generate the ultra-high-resolution hologram.

The overall block-diagram of the NLUT method is shown in Fig. S4. The 3D object can be treated as a set of images discretely sliced in the *z* direction. Each image at a fixed depth is approximated as a collection of emissive object points. In this method, only the fringe patterns of the center points on each image plane are pre-calculated by Eq. (S4.1) and stored in the memory, called principal fringe patterns (PFPs).

 (S4.1)

where *z_p_* is the depth of the center object point *P* (*x*, *y*, *z*_p_) and *φ*_p_ is the initial phase of the point *P*, *k* is the wave number defined as *k*=2π/*λ*, in which *λ* is the free-space wavelength of the light. Then, the fringe patterns for other object points on each image plane, called sub-computer generated holograms (sub-CGHs), can be obtained by simply shifting the pre-calculated PFPs according to the displaced values from the center to those points. The CGH of the 3D object can be generated by adding the sub-CGHs corresponding to all points. The calculated equation can be expressed as follows:

$I(x,y)=\sum_{p=1}^{N} a_{p}T(x-x_{p},y-y_{p},z_{p})$ (S4.2)

where *a*_p_ is the intensity of the point *P*, *x*_p_ and *y*_p_ are the horizontal and vertical coordinates, respectively. Then the 3D object is reconstructed.^1^


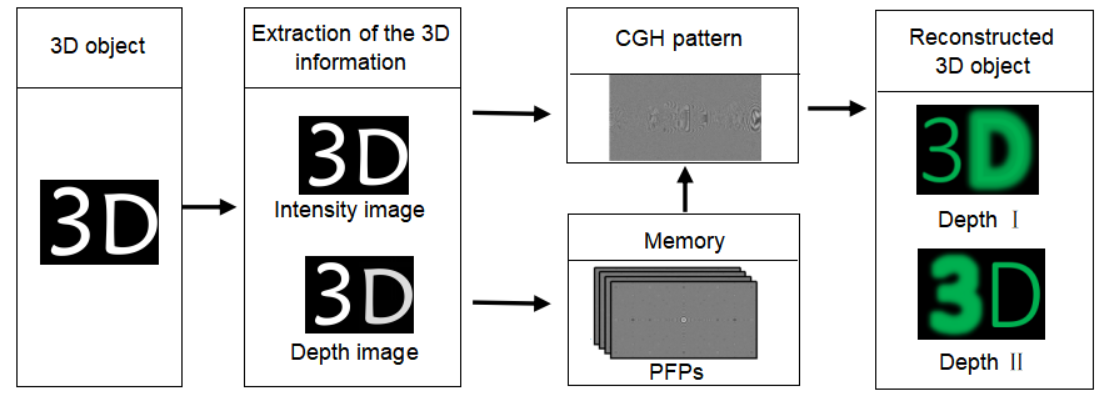


**Figure S4. Overall block-diagram of the NLUT method.**

Compared with the traditional point source model algorithm, the NLUT algorithm retains only simple addition operations, reduces the complexity of the calculation and therefore improves the calculation speed. However, when using ultra-high-resolution SLM and 3D objects, the amount of calculation will greatly increase and the calculation time of the hologram will become longer. Therefore, we improve the NLUT algorithm by optimizing the segmentation of the sub-hologram to further improve the calculation speed.^2^

The relationship between the recorded object, hologram, SLM and the viewing area of the reconstructed image is shown in Fig. S5a. The sizes of the SLM and hologram are both *H*, and the size of the object is *W*. *P* and *Q* are two endpoints of the object, corresponding to the *P*′ and *Q*′ of the reconstructed image respectively. The distance between the SLM and the reconstructed image is *L.* *R* is the viewing distance. The diffraction area of *Q*′ is *AC*, in which we can see the point endpoint *Q*′. Similarly, the diffraction space of *P*′ is *BD*, in which we can see the point endpoint *P*′. Thus, only in the overlapped area of *AC* and *BD* (the effective diffraction area *BC*), the complete reconstructed image can be observed. In the area *AB* and *CD*, only a part of the image can be observed. The regions of the sub-CGHs that make contribution to the effective diffraction area *BC* are called optimized diffraction areas (ODAs). On the basis of the effective diffraction area *BC*, each sub-CGH of the object point segmentation can be calculated and optimized.

To easily describe the optimized segmentation process, a cross section of the hologram reconstruction is selected (Fig. S5b). It is assumed that the SLM is located at *x* = 0. The boundary of the holographic diffracted light field is limited by the diffraction angle *α*.

$\alpha\leq\sin^{-1}\text{(}\frac{\lambda}{2p}\text{)}$ (S4.3)

where *p* is the pixel pitch of the SLM. The viewing angle *β* is twice the size of diffraction angle *α*.


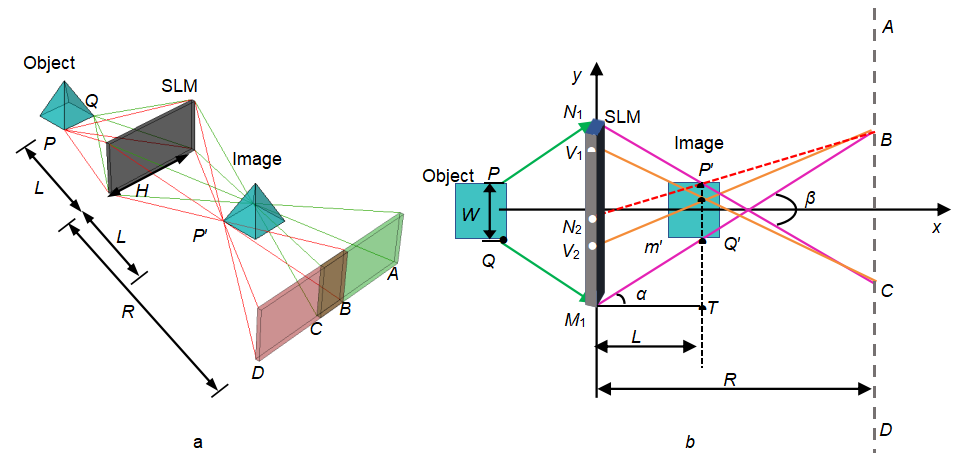


**Figure S5. Principle of the optimized segmentation algorithm. a** Relationship between the recorded object, hologram, SLM and the viewing area of the reconstructed image. **b** Optimized segmentation of the endpoint *P*′ and any point *m*′.

Apparently, the ODA of the sub-CGH corresponding to the endpoint *P*′ is the area *N*_1_*N*_2_. *m*′(*x*_0_, *y*_0_) is any point on the reconstructed image, and *V*_1_*V*_2_ is the ODA of the sub-CGH corresponding to the point *m*′. Wherever the point *m*′ is, the ODA of the point is corresponding to the effective diffraction area *BC*. Therefore, the ODA diffraction boundary *V*_1_(0, *y*_1_) and *V*_2_(0, *y*_2_) can be calculated by the geometric relationship, which are given by Eqs. (S4.4)-(S4.5),

$y_{1}=-\frac{H}{2}+\frac{R\text{(}2y_{0}+W\text{)}}{2\text{(}R-L\text{)}}$ (S4.4)

$y_{2}=\frac{H}{2}+\frac{R\text{(}2y_{0}-W\text{)}}{2\text{(}R-L\text{)}}$ (S4.5)

The ODA size *S* is the distance between *V*_1_(0, *y*_1_) and *V*_2_(0, *y*_2_), which can be calculated according to Eqs. (S4.4)-(S4.5).

$S=H-W\frac{R}{R-L}$ (S4.6)

Then, the exact position of the ODA in the sub-CGH is given by Eq. (S4.7):

$\tau=y_{2}-y_{0}=\frac{H}{2}+\frac{2Ly_{0}-WR}{2\text{(}R-L\text{)}}$ (S4.7)

where *τ* is the distance from the ODA coboundary to the sub-CGH center, which is related to the point position. Each sub-CGH is segmented according to the position and the size of the ODA. The position relationship of the two adjacent points ODAs can be calculated by Eq. (S4.8):

$v=\left(y_{2}'-y_{2}\right)p=\frac{R}{R-L}p$ (S4.8)

where *v* is the distance between two adjacent ODAs and *y*_2_′ is the ordinate of the adjacent point. Finally, the hologram is generated by the coherent superposition of all the ODAs.

**S5: Wide viewing angle holographic reproduction of objects with different resolutions**

The period of the tunable liquid crystal grating can be adjusted by controlling the *V*_DC_ and *V*_AC_ to achieve more flexible wide viewing angle holographic display. For objects with different resolutions, the viewing angle of the reproduced image is guaranteed to be continuous by adjusting the voltage of the liquid crystal grating. Here, a 2D object ‘flower’ with the resolution of 720×900 is used for the holographic reproduction, and the reconstructed distance is set to 15 cm. When *V*_DC_ = 4.0 V, *V*_AC_ = 4.5 V, the liquid crystal grating is in the large periodic order (the periodic pitch is 40 μm). At this time, the reconstructed image with continuous viewing angles can be seen, as shown in Figs. S6a-n. However, when the liquid crystal grating is in the small periodic order (*V*_DC_ = 3.7 V, *V*_AC_ = 0 V), there is a gap between the reproduced images, resulting in discontinuous viewing angles, which affects the viewing effect, as shown in Figs. S6 o-u.


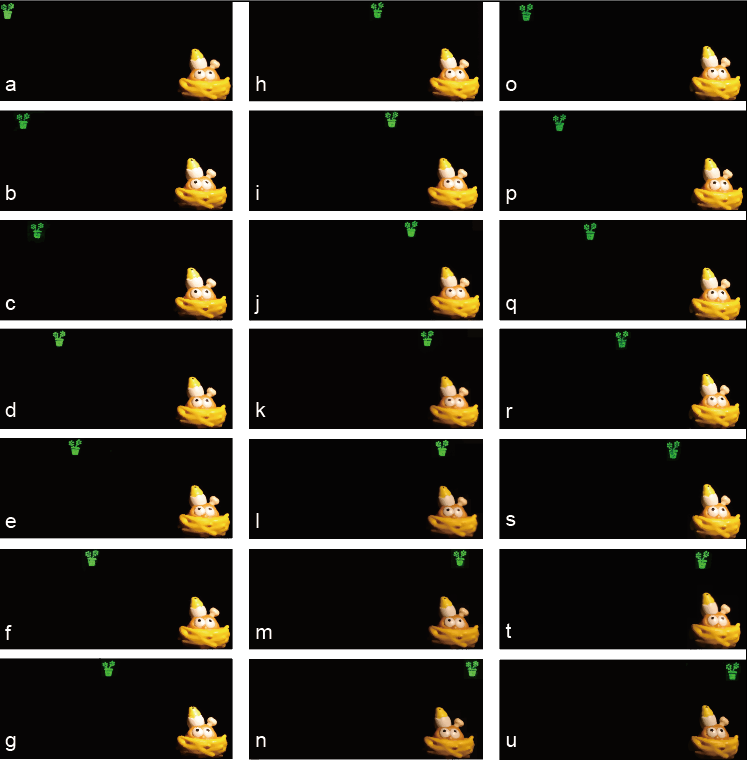


**Figure S6. Wide viewing reconstruction of the object ‘flower’. a-n** Reconstructed images when the liquid crystal grating is in the large periodic order. **o-u** Reconstructed images when the liquid crystal grating is in the small periodic order.

In our experiment, if the large periodic order is used for the object in Fig. 7, it will lead to overlapping of viewports in the reconstructed image, as shown in Fig. S7, which seriously affects the quality of the holographic reconstruction.

**
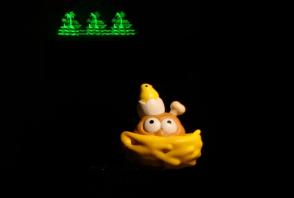
**

**Figure S7. Reconstructed image when the large periodic order is used for the object ‘island’.**

**References**

1. Kim, S. C., & Kim, E. S. Effective generation of digital holograms of three-dimensional objects using a novel look-up table method. *Applied Optics* **47**(19), D55-D62 (2008).
2. Li, Y. L. *et al*. Fast hologram generation method based on optimal segmentation of sub-computer-generated hologram. *Optics Express* **28**(21), 32185-32198 (2020).
