# Supplementary figures and images for: Tunable liquid crystal grating based holographic 3D display system with wide viewing angle and large size
Source: Light Sci Appl. 2022 Jun 21;11:188. doi: 10.1038/s41377-022-00880-y (PMC9213428; doi:10.1038/s41377-022-00880-y)

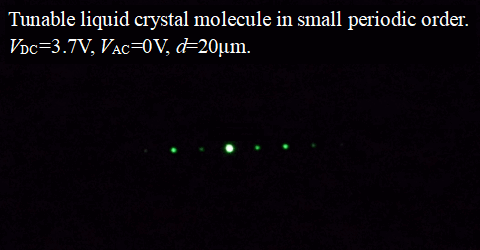

Supplement: Supplementary file 2 — Video m1 [file 41377_2022_880_MOESM2_ESM.gif]

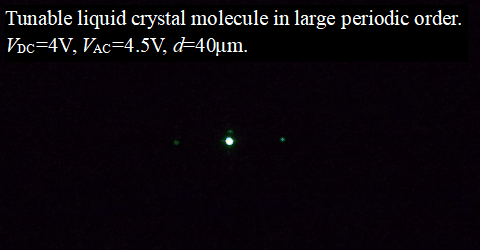

Supplement: Supplementary file 3 — Video m2 [file 41377_2022_880_MOESM3_ESM.gif]
